# A Time-Delay Low-Rank Reconstruction and Attention-BP-LSTM Framework for Axle-Box Bearing Temperature Prediction in Railway Vehicles

**DOI:** 10.3390/s26165137

**Published:** 2026-08-14

**Authors:** Yufei Xie, Kun Xie, Jinbai Zou, Wanyi Li, Yushuo Liu

**Affiliations:** School of Intellectual Technology, Shanghai Institute of Technology, Shanghai 201418, China; 18193140300@163.com (Y.X.); zoujb@sit.edu.cn (J.Z.); 17717086039@163.com (W.L.); 17596500511@163.com (Y.L.)

**Keywords:** axle-box bearing temperature prediction, high-speed train, Attention-BP-LSTM, low-rank sparse reconstruction, time-delay embedding, data preprocessing

## Abstract

The accuracy of axle-box bearing temperature prediction is important for monitoring the operating condition of high-speed electric multiple units. However, temperature data collected in service often contain missing values, abnormal fluctuations, and noise, which can affect the reliability of the prediction results. To address this problem, this paper proposes an Attention-BP-LSTM framework that combines data preprocessing with temperature prediction. First, missing data are completed by linear interpolation. The dynamically augmented temperature sequence is then processed using time-delay low-rank sparse decomposition to separate the normal temperature variation trend, sparse anomalies, and measurement noise, based on which the abnormal data are reconstructed. After Z-score normalization, the processed time series is converted into supervised learning samples using a sliding window. The prediction model consists of LSTM, an attention mechanism, Dropout, and a BP network. LSTM is used to extract the temporal dependencies in the temperature sequence, the attention mechanism assigns corresponding weights to different historical features, Dropout alleviates model overfitting, and the BP network completes the nonlinear mapping from temporal features to the predicted temperature. Experimental results based on axle-box bearing temperature data show that, compared with BP, LSTM, and BP-LSTM, the proposed model achieves lower MAE and RMSE and a higher R^2^. This indicates that improving data quality before prediction, together with attention-based temporal feature extraction, helps improve the reliability of short-term axle-box bearing temperature prediction.

## 1. Introduction

In railway systems, axle-box bearings in high-speed trains play a critical role in ensuring operational safety and service efficiency. As key components of high-speed train bogies, axle-box bearings provide essential support for rotating mechanical assemblies [1,2]. Although their failure probability remains relatively low as high-speed trains become more widely deployed, a single bearing failure may lead to severe consequences. Unlike ordinary industrial bearings, axle-box bearings operate for long periods under harsh conditions, including high speed, heavy load, strong environmental variation, and limited accessibility for manual inspection. Without effective monitoring, these bearings are prone to premature failure [3,4]. Therefore, effective condition monitoring of axle-box bearings has strong practical significance, since it enables abnormal states to be identified before faults develop into severe failures. Among various measurable parameters, bearing temperature is a core condition indicator because it directly reflects the thermal response of the bearing system under different operating conditions and fault states. Existing studies show that high-speed trains are commonly equipped with temperature sensors to monitor bearing operating conditions, and the temperature variation in axle-box bearings is closely related to train speed, fault type, fault severity, and external environmental factors [5]. In actual railway operation, temperature-based monitoring is also widely used in hot axle detection systems. These systems collect bearing temperature data through infrared sensors and automatically trigger early warnings once abnormal temperature rise is detected [6]. Predictive maintenance strategies supported by advanced data analysis and machine learning have become important means of improving the operational reliability of such components.

With the development of railway condition monitoring technologies, research on axle-box bearing health assessment gradually shifts from passive fault alarming to predictive condition evaluation. Traditional monitoring systems mainly use wayside or on-board sensing devices to collect abnormal temperature, acoustic, and vibration response signals from bearings. Existing studies summarize fault diagnosis technologies for high-speed train axle-box bearings from the perspectives of detection instruments, sensor deployment, and diagnostic methods. Among them, both wayside monitoring and on-board monitoring schemes receive extensive attention, and vibration-based, acoustic-based, and temperature-based detection methods are widely used to identify abnormal states of axle-box bearings [7]. Acoustic detection methods, such as wayside acoustic detection systems, can identify early bearing faults by analyzing acoustic signals generated by train bearings [8]. Vibration and acoustic emission signals are also widely used in fault diagnosis studies of axle-box bearings. Vibration analysis is one of the most commonly used methods for bearing fault detection, while acoustic emission technology, owing to its high-frequency characteristics and high sensitivity, is suitable for condition monitoring of high-speed train wheelset bearings [9].

However, conventional threshold-based temperature alarms only determine whether the measured axle-box temperature exceeds a preset limit, and they can hardly reflect the subsequent temperature variation trend of bearings under varying operating conditions. In recent years, research on train axle-box bearing condition monitoring and axle temperature prediction has gradually shifted from traditional sensor-based monitoring to data-driven methods and intelligent early warning strategies. Bhadouria et al. reviewed recent advances in railway condition monitoring and fault diagnosis from the perspective of predictive maintenance, emphasizing that condition monitoring can support the early detection of developing faults and remaining-life assessment for critical railway assets such as wheelsets, axles, bearings, and tracks [10]. Zheng et al. established a thermal model for high-speed train axle-box bearings considering lubrication-state evolution and vehicle dynamic response. By introducing the bearing loads obtained from an axle-box-bearing–vehicle dynamics model, they analyzed the temperature distribution of axle-box bearings under different operating conditions and validated the model using long-term wireless temperature-monitoring data [11]. Yang et al. construct a high-speed train bearing temperature fault detection model that integrates physical mechanisms with data-driven methods. By combining an axle-box thermal behavior model with neural networks such as BP and LSTM, the model realizes healthy temperature prediction and fault identification of bearings under service conditions [12]. To address insufficient abnormal samples, missing values, and outliers in high-speed train axle-box temperature alarm systems, Li et al. propose an axle temperature monitoring and alarm model that integrates dynamic principal component preprocessing, GAN-based data generation, and an optimized Transformer, which improves the recognition capability for high-temperature axle alarm levels [13]. Li et al. further propose a spatio-temporal prediction method for locomotive axle temperature based on an integrated graph convolutional recurrent unit network. This method uses GCN to extract spatial correlations among different axle positions and combines GRU, BiLSTM, and an ensemble optimization strategy to improve axle temperature prediction accuracy [14]. Luo et al. developed a thermal analysis model for axle-box bearings under wheel flat excitation by coupling the vehicle operation environment with the heat transfer process. Their results show that the axle-box bearing temperature increases with vehicle speed and wheel flat length, providing useful guidance for bearing condition monitoring and maintenance under complex service excitations [15].

Studies show that axle-box bearing temperature data exhibit clear nonlinearity, time-varying behavior, and hysteresis, and are also affected by operating speed, ambient temperature, load condition, track condition, and sensor acquisition quality. In practical monitoring data, missing values, abnormal sudden changes, and noise disturbances often occur. If raw data are directly fed into the prediction model, the model may learn incorrect local features, which reduces prediction accuracy and generalization ability [16]. Therefore, it is necessary to build a prediction framework that can both improve the quality of raw axle temperature data and capture the nonlinear dynamic relationship between historical temperature data and the future operating state of bearings. Based on this research background, this paper integrates an improved data preprocessing method and constructs an Attention-BP-LSTM prediction model to improve the accuracy and reliability of high-speed train axle temperature prediction.

The main contributions of this paper are as follows. First, an abnormal data reconstruction method is proposed for high-speed train axle-box bearing temperature data. This method constructs a dynamic augmented matrix through time-delay embedding and uses low-rank sparse decomposition to separate the normal temperature variation pattern from sparse abnormal disturbances, thereby improving the quality of input data. Second, a BP-LSTM axle temperature prediction model integrating the attention mechanism and Dropout regularization is constructed. LSTM is used to extract temporal dependence features from temperature sequences, the attention mechanism is used to enhance the representation of key historical information, and the BP regression layer is used to complete nonlinear temperature mapping. Third, model comparison experiments are conducted to verify the effectiveness of the proposed method, demonstrating that abnormal data reconstruction, the attention mechanism, and Dropout regularization all contribute to improving axle temperature prediction accuracy and generalization performance.

## 2. Data Preprocessing

### 2.1. Missing Data Handling

During the acquisition of axle-box bearing temperature data, a small number of missing values may occur due to sensor communication interruption, acquisition delay, or abnormal data storage. Considering that axle temperature data from high-speed trains usually have dense sampling, strong temporal continuity, and smooth variation between adjacent sampling instants, this paper uses linear interpolation to complete the missing values.

Let the original temperature sequence of a bearing measuring point be $T=\{t_{1},t_{2},\cdots \;,t_{N}\}$, with the corresponding sampling instants denoted as $\tau_{1},\tau_{2},\cdots \;,\tau_{N}$. For a single missing value $t_{i}$, its value can be estimated using the valid data points before and after it:

(1) $${\hat{t}}_{i}=t_{i-1}+\frac{t_{i+1}-t_{i-1}}{\tau_{i+1}-\tau_{i-1}}\left(\tau_{i}-\tau_{i-1}\right)$$

When the missing value appears at the beginning or the end of the sequence, the nearest valid value is used for filling, namely ${\hat{t}}_{1}=t_{2},{\hat{t}}_{N}=t_{N-1}$.

For the case where multiple consecutive data points are missing, assume that m data points from $\tau_{k}$ to $\tau_{k+m-1}$ are missing, and that the nearest valid data points before and after this missing segment are $t_{k-1}$ and $t_{k+m}$, respectively. Then the estimated value of the j-th missing point can be expressed as:

(2) $${\hat{t}}_{k+j}=t_{k-1}+\frac{t_{k+m}-t_{k-1}}{\tau_{k+m}-\tau_{k-1}}\left(\tau_{k+j}-\tau_{k-1}\right),\;0\leq j<m$$

**Figure 1:**
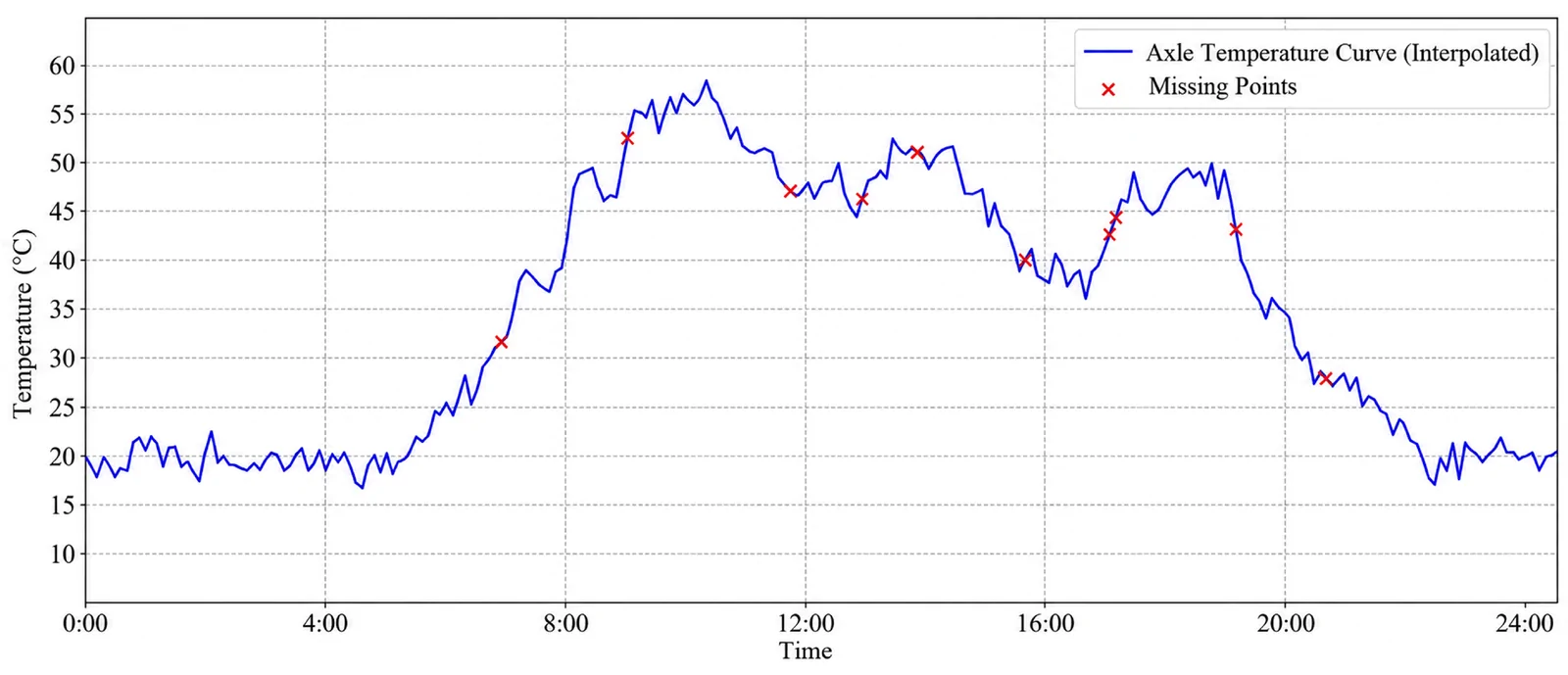
Results of Missing Value Imputation for Axle Temperature Data.

The missing-value imputation result is shown in Figure 1. It can be observed that linear interpolation smoothly completes the missing positions, and the imputed axle temperature sequence preserves the overall variation trend and temporal continuity of the original data.

### 2.2. Outlier Detection and Reconstruction

Outliers in axle-box bearing temperature data may arise from transient sensor faults, communication noise, external environmental interference, or abnormalities in the data acquisition system. Although such abnormal points account for only a small proportion of the whole sequence, they can disturb the temporal structure of the data and degrade the training performance of subsequent prediction models. Therefore, outliers need to be identified and corrected before model construction. Considering the strong dynamic correlation of axle-box bearing temperature sequences and the sparse distribution of outliers, this paper adopts a low-rank sparse decomposition method based on time-delay embedding for abnormal data reconstruction. The method decomposes the data matrix into a low-rank component, sparse outliers, and a noise component, and then reconstructs and corrects the outliers through optimization [17]. Let the axle-box bearing temperature data matrix after missing-value processing be $X_{s}\in R^{N\times m}$, where N denotes the number of sample points and m denotes the number of variables. To capture the dynamic temporal correlation of the data, a delay parameter d is introduced, and the original data matrix is expanded into a time-delay augmented matrix:

(3) $$X_{d}={\left[X_{s}(1:N-d+1,1:m)X_{s}(2:N-d+2,1:m)\cdots X_{s}(d:N,1:m)\right]}^{T}$$

where $x_{i}\in R^{1\times m}$ denotes the multivariate axle temperature observation at the i-th sampling instant. Here, d denotes the number of time delays, also referred to as the embedding dimension, while N and m represent the number of observations and the number of original variables, respectively. Therefore, the augmented matrix $X_{d}$ contains d delayed versions of the original data and has a dimension of $dm\times \left(N-d+1\right)$.

Based on the time-delay augmented matrix, the decomposition is formulated as:

(4) $$X_{d}=L+S+E$$

where L is a low-rank matrix representing the normal variation pattern of axle temperature data, S is a sparse matrix corresponding to outliers, and E is a random noise term. This decomposition process can be expressed as the following optimization problem:

(5) $$\underset{L,S}{min}∥L{∥}_{*}+\lambda ∥S{∥}_{1}s.t.∥X_{d}-L-S{∥}_{F}\leq \varepsilon$$

where $∥L{∥}_{*}$ denotes the nuclear norm, which constrains the low-rank property, $∥S{∥}_{1}$ denotes the $l_{1}$ norm, which constrains the sparse property, λ is a balance parameter, it was determined using the dimension-dependent setting $\lambda =\frac{1}{\sqrt{\max\left(\mathrm{dm},N-d+1\right)}},$ where dm and $\left(N-d+1\right)$ are the row and column dimensions of the time-delay augmented matrix $X_{d}$, respectively. Based on the dimensions of $X_{d}$ in the present dataset, λ was set to 0.0144 and remained fixed throughout the decomposition process, and ε is the noise tolerance, which was set to 26.28.

After the sparse outlier matrix S is obtained, the corrected augmented matrix can be written as $X_{r}=X_{d}-S$. The augmented matrix is then restored to the original time-series matrix through an inverse mapping operation:

(6) $${\hat{X}}_{s}=R\left(X_{r}\right)=R\left(X_{d}-S\right)$$

where R(⋅) denotes the restoration operation from the time-delay augmented matrix to the original time-series matrix.

**Figure 2:**
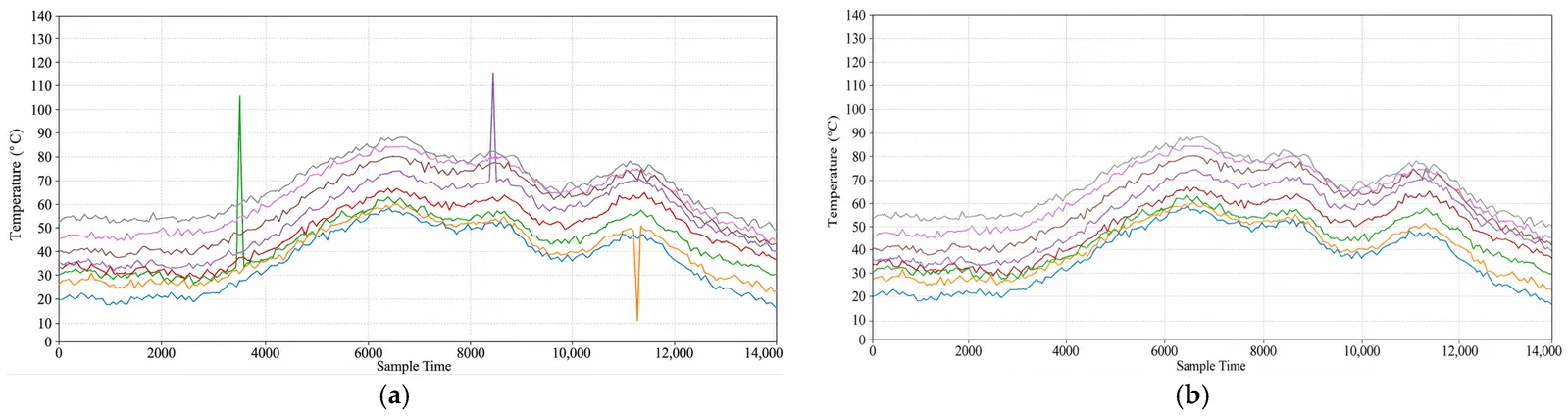
(**a**) Axle Temperature Data before Outlier Processing. (**b**) Axle Temperature Data after Outlier Processing.

The results before and after outlier processing are shown in Figure 2a and Figure 2b, respectively. The curves of different colors in the figure correspond to different temperature measuring points of the axle box bearing respectively. It can be observed that transient spikes and abnormal sudden drops in the original axle temperature sequence are effectively corrected. The processed curve becomes smoother while preserving the overall variation trend and temporal evolution characteristics of the original data.

### 2.3. Normalization Processing

To eliminate dimensional differences among different variables and improve the stability of model training, this paper applies Z-score normalization to the corrected axle temperature data. Let the preprocessed data matrix be $X\in R^{N\times m}$. For the j-th variable, its mean and standard deviation are defined as follows:

(7) $$\mu_{j}=\frac{1}{N}\sum_{i=1}^{N}x_{ij}$$

(8) $$\sigma_{j}=\sqrt{\frac{1}{N}\sum_{i=1}^{N}(x_{ij}-\mu_{j}{)}^{2}}$$

The standardized data are then obtained as:

(9) $$z_{ij}=\frac{x_{ij}-\mu_{j}}{\sigma_{j}}$$

After this processing, all variables are transformed into dimensionless data, which reduces the influence of scale differences on model training. The resulting standardized matrix serves as the input for subsequent feature extraction and prediction model construction.

### 2.4. Data Processing Pipeline

To avoid data leakage, the axle-box bearing temperature sequence was kept in its original time order during data preparation. Before any preprocessing step, the raw time-series data were first divided into a training part and an independent test set at a ratio of 70:30. No random shuffling was used. Then, 10% of the training part was used as the validation set to monitor the training process and apply early stopping, while the remaining 90% was used for model training.

After the data split, missing-value interpolation and outlier reconstruction were carried out separately in the training subset, validation set, and test set. In other words, the preprocessing of the training subset did not use any data from the validation set or the test set.

For Z-score normalization, the mean and standard deviation were calculated only from the training subset. The same values were then used to normalize the validation set and the test set. The inverse transformation after prediction was also based on the mean and standard deviation of the training subset.

The sliding-window samples were built after interpolation, outlier reconstruction, and normalization. The window length was set to *p* = 5. Samples were generated separately within the training subset, validation set, and test set, and no window crossed the boundary between different data sets. Therefore, interpolation, low-rank reconstruction, normalization, and sliding-window construction were all performed after the data split, which prevented information from the validation set or test set from entering the model training process.

## 3. Prediction Model

### 3.1. BP Model Structure

**Figure 3:**
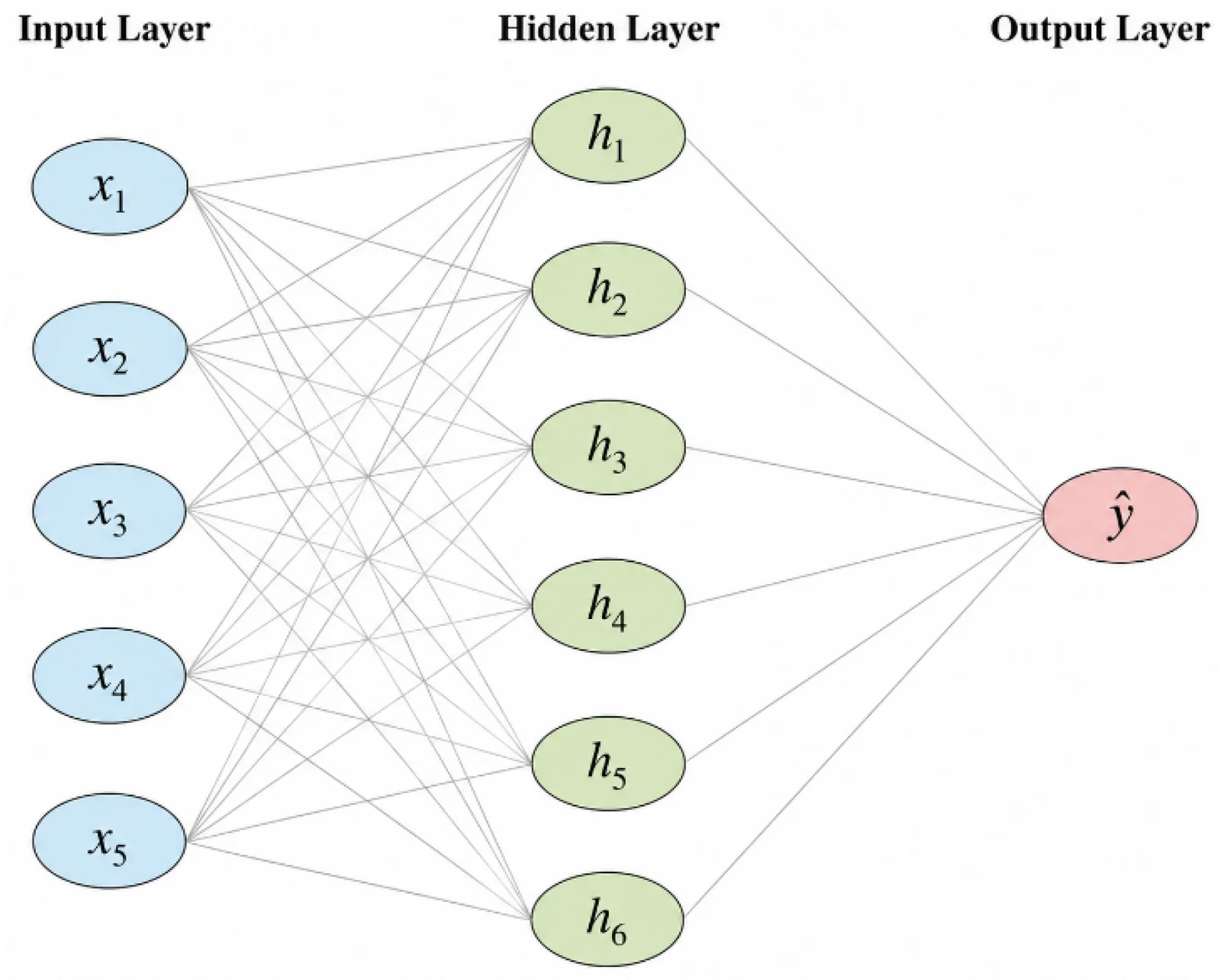
Schematic Diagram of The BP Neural Network.

The BP neural network is a typical feedforward neural network that can model the nonlinear relationship between input features and output targets in a data-driven manner. Existing studies apply it to temperature modeling and fault detection of high-speed train axle-box bearings, which verifies its applicability in axle temperature state representation [18]. The BP temperature prediction model developed in this study adopts a feedforward neural network architecture consisting of an input layer, two hidden layers, and an output layer. The numbers of neurons in the two hidden layers are set to 64 and 32, respectively, and the network architecture is illustrated in Figure 3. In all subsequent experiments, supervised learning samples are constructed from historical axle box bearing temperature sequences, and the number of input nodes is determined by the sliding window length.

The hidden layers employ the ReLU activation function to enhance the nonlinear representation capability and accelerate training convergence. Its expression is given by:

(10) f(x)=max(0,x)

The output layer uses a linear activation function to satisfy the requirement for continuous-valued temperature prediction, allowing the model to directly estimate the bearing temperature at the next time step.

The nonlinear relationship between the input features and the predicted temperature is established through a multilayer fully connected network. The forward propagation process is expressed as:

(11) $$\hat{y}=F(x;\Theta )$$

where x denotes the input feature vector, $\hat{y}$ denotes the predicted temperature, F(⋅) represents the nonlinear mapping established by the BP neural network, and Θ denotes all trainable weights and bias parameters.

Since the prediction target is a continuous temperature value, the mean squared error (MSE) is adopted as the loss function to measure the discrepancy between the predicted and actual temperatures. It is defined as:

(12) $$E=\frac{1}{N}\sum_{i=1}^{N}(y_{i}-{\hat{y}}_{i}{)}^{2}$$

where N is the number of samples, $y_{i}$ is the actual temperature of the i th sample, and ${\hat{y}}_{i}$ is the corresponding predicted temperature.

### 3.2. LSTM Network

LSTM is an improved structure of recurrent neural networks. It regulates the retention and forgetting of historical information through memory cells and gating mechanisms, and can effectively capture long-term dependence in time series [19]. The LSTM cell mainly consists of a forget gate, an input gate, an output gate, and a memory cell. At each time step, the forget gate determines which historical information should be retained, while the input gate controls the amount of new information stored in the memory cell. Finally, the output gate determines the hidden state passed to the next time step.

The forget gate is defined as

(13) $$f_{t}=\sigma (W_{f}[h_{t-1},x_{t}]+b_{f})$$

The input gate is calculated by

(14) $$i_{t}=\sigma (W_{i}[h_{t-1},x_{t}]+b_{i})$$

The candidate memory state is

(15) $${\tilde{C}}_{t}=tanh(W_{c}[h_{t-1},x_{t}]+b_{c})$$

The cell state is updated as

(16) $$C_{t}=f_{t}⊙C_{t-1}+i_{t}⊙{\tilde{C}}_{t}$$

The output gate is

(17) $$o_{t}=\sigma (W_{o}[h_{t-1},x_{t}]+b_{o})$$

Finally, the hidden state is

(18) $$h_{t}=o_{t}⊙tanh(C_{t})$$

where W and b denote the weight matrices and bias vectors, respectively, σ(⋅) represents the sigmoid activation function, and ⊙ denotes element-wise multiplication.

Compared with the BP neural network, the LSTM network can effectively exploit temporal correlations within sequential temperature data, making it more suitable for bearing temperature prediction. Nevertheless, the predictive performance of LSTM is highly dependent on parameter initialization and optimization. In practical applications, inappropriate initial weights may lead to slow convergence or convergence to local optima, thereby limiting the prediction accuracy.

### 3.3. BP-LSTM Model

**Figure 4:**
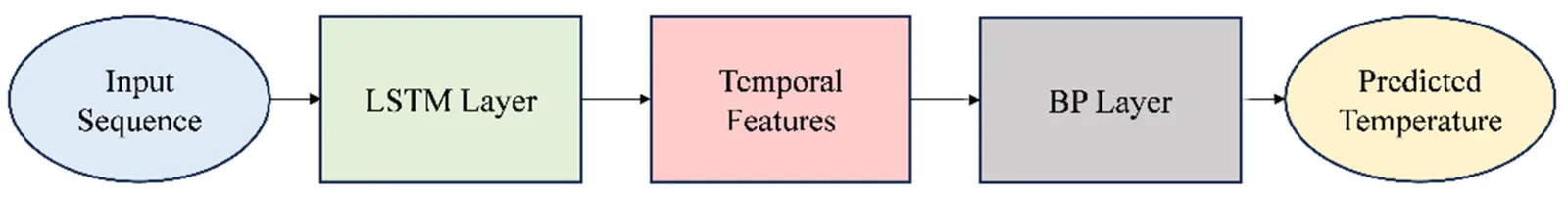
Schematic Diagram of The BP-LSTM Temperature Prediction Model.

To enhance the optimization capability of the LSTM model, this study combines the BP optimization strategy with the LSTM network to construct a BP-LSTM prediction model. In the proposed framework, the BP algorithm is employed to iteratively optimize the network parameters by minimizing the prediction error through gradient backpropagation. By continuously updating the weights and biases during training, the BP-LSTM model achieves faster convergence and improved prediction stability. The overall structure of the proposed model is shown in Figure 4.

Let the input historical temperature sequence be

(19) $$X_{i}=x_{i-w+1},x_{i-w+2},\cdots \;,x_{i}$$

where w denotes the input window length. After the input sequence is processed by the LSTM layer, the hidden state sequence is obtained as $H=\{h_{1},h_{2},\cdots \;,h_{T}\}$, where T is the length of the input sequence and $h_{t}$ is the hidden state corresponding to the t-th time step. The attention layer then calculates the weight of each hidden state according to its importance to the prediction task and generates a weighted feature representation. Finally, the BP regression layer outputs the predicted temperature based on this feature:

(20) $${\hat{y}}_{i}=f_{BP}\left(F_{i}\right)$$

where $F_{i}$ denotes the temporal feature extracted by the LSTM network and the attention mechanism, $f_{BP}(\cdot )$ represents the nonlinear mapping established by the BP network, and ${\hat{y}}_{i}$ denotes the predicted value of the model.

### 3.4. Attention-BP-LSTM Model

In the basic BP-LSTM model, LSTM can extract temporal features from the temperature sequence, but different historical time steps do not contribute equally to future temperature variation. If only the hidden state at the final time step is used, or if all hidden states are treated with equal weights, the information from key time steps may be ignored. Therefore, this paper introduces an attention mechanism after the LSTM layer, allowing the model to adaptively assign contribution weights to different time steps.

#### 3.4.1. Mechanism

For the hidden state sequence output by LSTM, $H=\{h_{1},h_{2},\cdots \;,h_{T}\}$, the attention score of each time step is first calculated as:

(21) $$e_{t}=v^{T}tanh\left(W_{h}h_{t}+b_{h}\right)$$

Then, the Softmax function is used to normalize the scores and obtain the attention weights:

(22) $$\alpha_{t}=\frac{exp(e_{t})}{\sum_{t=1}^{T}exp(e_{t})}$$

where $\alpha_{t}$ denotes the contribution degree of the t-th time step to the prediction result and satisfies:

(23) $$\sum_{t=1}^{T}\alpha_{t}=1$$

Finally, the hidden states of all time steps are weighted and summed to obtain the context feature vector:

(24) $$F_{i}=\sum_{t=1}^{T}\alpha_{t}h_{t}$$

This vector provides an integrated representation of the key temporal information in the input sequence and serves as the input feature of the BP network. Through this mechanism, the model can assign higher weights to intervals with evident temperature fluctuations or strengthened variation trends, thereby improving its ability to represent short-term temperature changes.

In addition, to reduce the risk of overfitting under limited sample conditions, this paper adds Dropout regularization between the attention layer and the BP output layer. During training, Dropout randomly masks part of the neuron outputs with a certain probability, so that the model learns parameters under a randomly sparsified network structure. This helps alleviate overfitting and improve model generalization. During testing, Dropout is disabled, and the complete network is used for prediction [20]. In this paper, the Dropout rate is set to 0.3 to improve model generalization and prediction stability.

#### 3.4.2. Network Architecture

**Figure 5:**
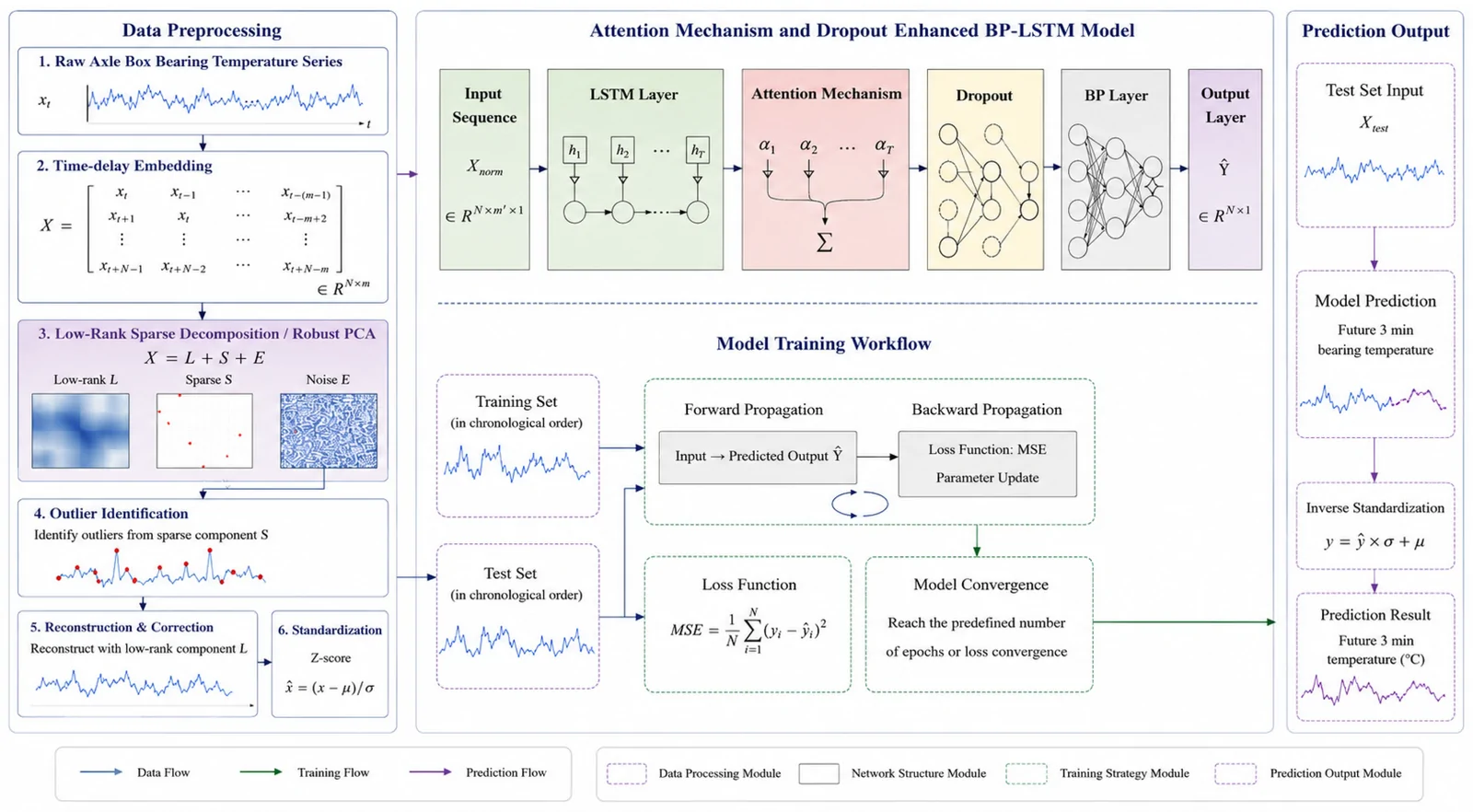
Schematic Diagram of The Attention-BP-LSTM Temperature Prediction Model.

The architecture of the proposed Attention-BP-LSTM model is illustrated in Figure 5. The model integrates the temporal feature extraction capability of LSTM, the parameter optimization ability of BP, and the adaptive feature weighting mechanism of Attention into a unified prediction framework.

First, the historical axle-box bearing temperature sequence is reconstructed into supervised learning samples using a sliding-window strategy and normalized before being fed into the network. The LSTM layer is employed to extract temporal dependencies from the input sequence, generating a series of hidden state vectors that characterize the dynamic evolution of bearing temperature.

Subsequently, the attention mechanism is introduced to evaluate the relative importance of different hidden states. By assigning adaptive attention weights to each time step, the model selectively emphasizes informative temporal features while suppressing redundant or less relevant information. The weighted hidden representations are then aggregated into a context vector that contains the most discriminative information for temperature prediction.

Finally, the context vector is transmitted to the fully connected output layer, where the BP algorithm iteratively updates the network parameters through error backpropagation to minimize the prediction loss. Compared with the conventional BP-LSTM model, the proposed architecture not only captures long-term temporal dependencies but also focuses on the most informative historical observations, thereby improving prediction accuracy and robustness under varying operating conditions.

#### 3.4.3. Model Training Process

After data preprocessing is completed, this paper constructs supervised learning samples using the sliding-window method. The axle-box bearing temperature sequence over the historical w time steps is used as the model input, and the temperature at the next time step is used as the prediction output. The sample set can be expressed as:

(25) $$D=\{(X_{i},y_{i}){\}}_{i=1}^{n}$$

where $X_{i}$ denotes the i-th input sample, $y_{i}$ denotes the corresponding actual temperature value, and n denotes the number of samples. The model prediction process can be expressed as:

(26) $${\hat{y}}_{i}=F\left(X_{i};\theta \right)$$

where F(⋅) denotes the Attention-BP-LSTM model constructed in this paper, and θ denotes all trainable parameters in the model.

This paper uses the mean squared error as the loss function:

(27) $$Loss=\frac{1}{n}\sum_{i=1}^{n}(y_{i}-{\hat{y}}_{i}{)}^{2}$$

where $y_{i}$ and ${\hat{y}}_{i}$ denote the actual temperature value and the predicted temperature value, respectively. The model parameters are iteratively updated using the Adam optimization algorithm to improve training convergence efficiency and stability.

To avoid information leakage caused by random splitting of time-series data, this paper divides the training set, validation set, and test set in chronological order. The training set is used for model parameter learning, the validation set is used for hyperparameter adjustment and convergence assessment, and the test set is used only for final prediction performance evaluation. The Z-score normalization parameters are calculated only from the training set and are then applied to the validation set and test set. After the model outputs the standardized prediction value, inverse normalization is performed to restore it to the actual temperature scale:

(28) $${\hat{y}}_{real}=\hat{y}\sigma_{y}+\mu_{y}$$

where ${\hat{y}}_{real}$ denotes the predicted value under the actual temperature scale, $\hat{y}$ denotes the standardized predicted value, and $\mu_{y}$ and $\sigma_{y}$ denote the mean and standard deviation of the prediction target variable in the training set, respectively.

The main parameter settings of the proposed model are shown in Table 1.

Through the above training process, the Attention-BP-LSTM model can extract key temporal features from historical axle temperature sequences and achieve short-term prediction of axle-box bearing temperature at future time steps. Compared with a single BP model, the proposed model can better exploit the temporal dependence of temperature sequences. Compared with a basic LSTM model, the attention mechanism and BP regression layer further enhance the model’s ability to represent key historical information and nonlinear temperature-rise patterns.

## 4. Results and Discussion

### 4.1. Experimental Setup

To validate the effectiveness of the proposed Attention-BP-LSTM model in predicting axle box bearing temperatures on high-speed trains, this paper used 10 consecutive days of in-service axle-box bearing temperature monitoring data for scenario-based validation. The sampling interval was 3 min, and the dataset contained 4800 original chronological temperature records. The data covered multiple typical operating conditions, including train stabling, depot departure and acceleration, mainline operation, and depot return for inspection, thereby reflecting the dynamic temperature characteristics of axle-box bearings under different operating scenarios. As described in Section 2.4, the original chronological sequence was first divided into training and test sets before interpolation, low-rank reconstruction, normalization, and sliding-window construction were performed. This ensured that no information from the validation or test sets was used during training-set preprocessing. Considering the strong temporal dependence of axle-box bearing temperature, a sliding-window strategy was adopted to construct supervised learning samples, with a window length of p=5. The five historical temperature values preceding the current time step were used as input to predict the axle box bearing temperature for the next 3 min.

To evaluate the prediction performance of different models, the BP, LSTM, BP-LSTM, and Attention-BP-LSTM models were tested using the same axle-box bearing temperature dataset. The same data preprocessing strategy, sliding-window configuration, and training and testing samples were adopted to ensure the comparability of the experimental results. The prediction results and corresponding errors of the different models were analyzed comparatively. The BP model is configured with two hidden layers, containing 64 and 32 neurons, respectively; the LSTM model contains 64 hidden units; the BP-LSTM model consists of an LSTM feature extraction layer combined with a BP regression layer; the Attention-BP-LSTM model builds upon the BP-LSTM by additionally incorporating an attention mechanism and Dropout regularization with a Dropout ratio of $p_{d}=0.3$. All models were trained using the Adam optimizer with a learning rate of 0.001, and the mean squared error was used as the loss function.

To quantitatively evaluate the prediction performance of different models, this paper selects root mean square error (RMSE), mean absolute error (MAE), and coefficient of determination (R^2^) as evaluation metrics. Their calculation formulas are as follows:

(29) $$RMSE=\sqrt{\frac{1}{n}\sum_{i=1}^{n}(y_{i}-{\hat{y}}_{i}{)}^{2}}$$

(30) $$MAE=\frac{1}{n}\sum_{i=1}^{n}|y_{i}-{\hat{y}}_{i}|$$

(31) $$R^{2}=1-\frac{\sum_{i=1}^{n}(y_{i}-{\hat{y}}_{i}{)}^{2}}{\sum_{i=1}^{n}(y_{i}-\bar{y}{)}^{2}}$$

where $y_{i}$ denotes the actual temperature, ${\hat{y}}_{i}$ denotes the predicted temperature, and $\bar{y}$ denotes the mean value of the actual temperature.

### 4.2. Comparison with Different Prediction Models

**Figure 6:**
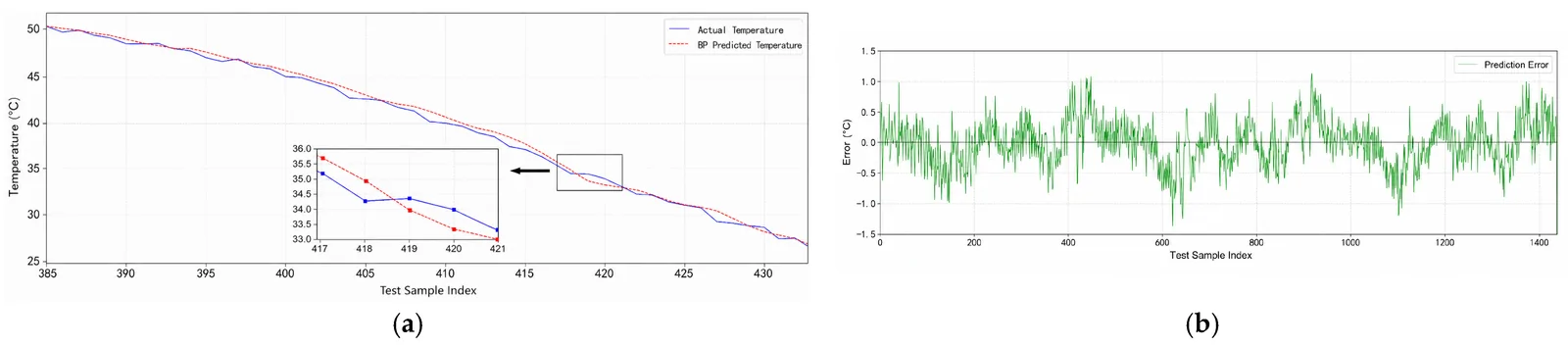
(**a**) Comparison of Predicted and Actual Local Temperatures on The BP Neural Network Test Set. (**b**) Prediction Error of Bearing Temperature Based on The BP Neural Network.

The BP neural network was first employed as the basic nonlinear prediction model. Figure 6a compares the BP-predicted temperature with the actual axle-box bearing temperature, while Figure 6b presents the corresponding prediction errors. The BP model can generally reproduce the overall variation tendency of bearing temperature. However, deviations between the predicted and actual values can still be observed at some sampling points, indicating that the feedforward network has limited ability to capture the temporal dependence of the temperature sequence.

**Figure 7:**
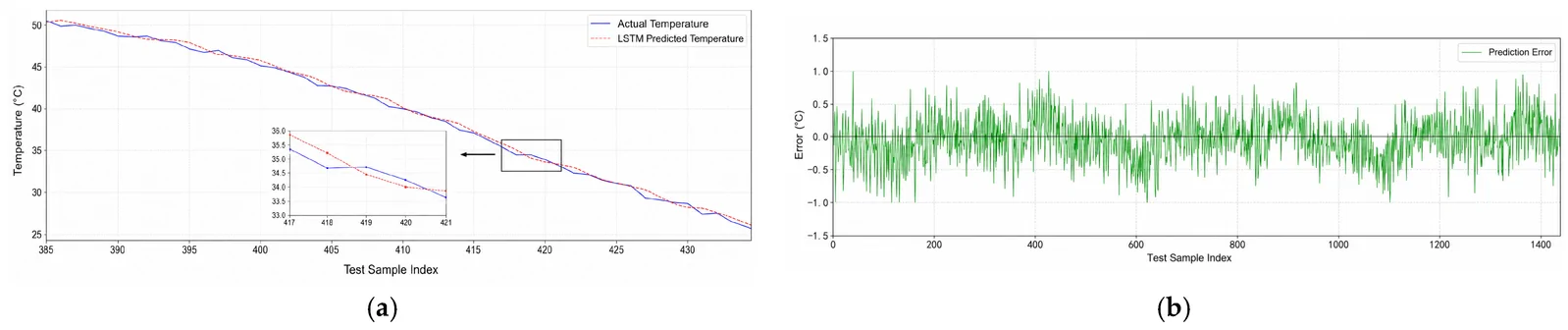
(**a**) Comparison of Predicted and Actual Local Temperatures on The LSTM Neural Network Test Set. (**b**) Prediction Error of Bearing Temperature Based on The LSTM Neural Network.

To further exploit the temporal characteristics of axle-box bearing temperature, the LSTM model was applied to the same prediction task. The corresponding prediction results and errors are illustrated in Figure 7. Compared with the BP model, the LSTM prediction curve follows the actual temperature variation more closely because its recurrent structure and gating mechanism enable historical temperature information to be retained and transmitted over time. As shown in Figure 7b, the prediction errors are also reduced, demonstrating the importance of temporal feature extraction for axle-box bearing temperature prediction.

**Figure 8:**
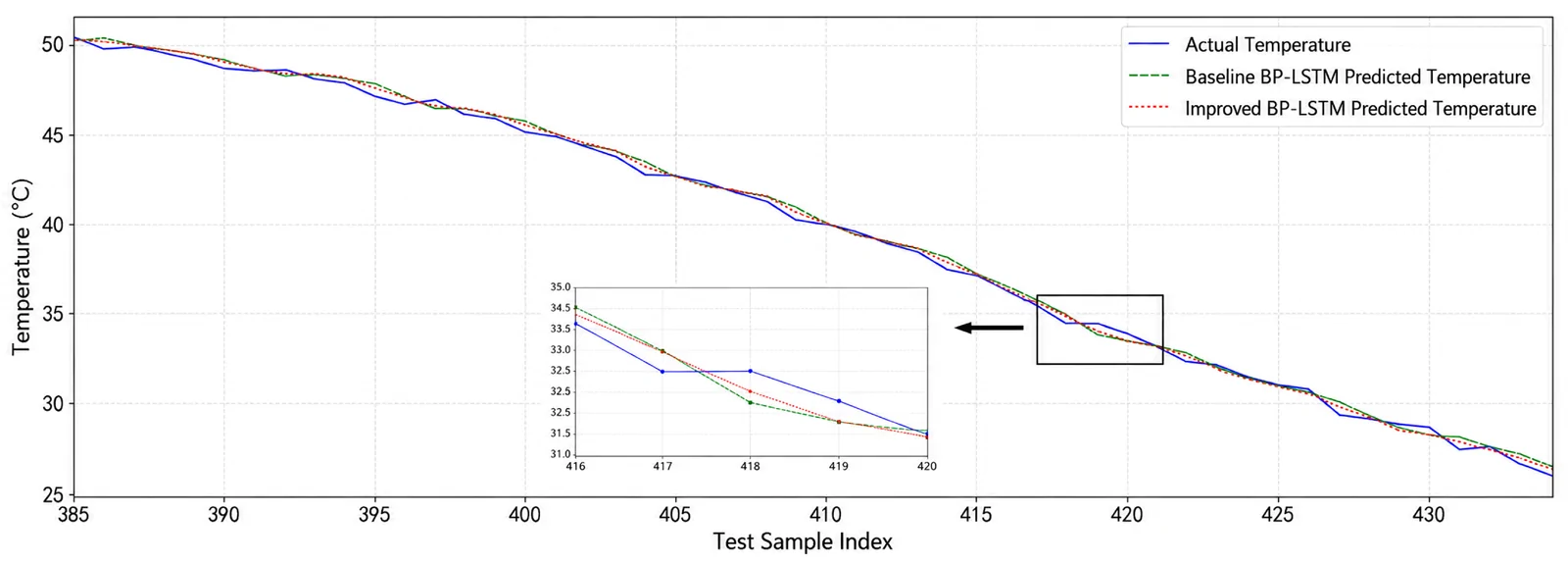
Comparison of Local Temperature Prediction Performance between The Basic BP-LSTM Model and The Attention-BP-LSTM Model.

Based on the above results, the basic BP-LSTM model was further constructed by combining the nonlinear mapping capability of BP with the temporal feature extraction capability of LSTM. To more clearly evaluate the contribution of the attention mechanism, the prediction results of the basic BP-LSTM and the Attention-BP-LSTM models are directly compared in Figure 8. It can be observed that both models can track the overall variation trend of axle-box bearing temperature, and their predicted curves show high consistency with the actual curve. Compared with the basic BP-LSTM model, the prediction results of Attention-BP-LSTM are closer to the actual temperature values, especially in intervals where local temperature changes occur rapidly. This indicates that the attention mechanism enhances the ability of the model to extract features from key historical time steps, while Dropout regularization also helps improve prediction stability.

**Figure 9:**
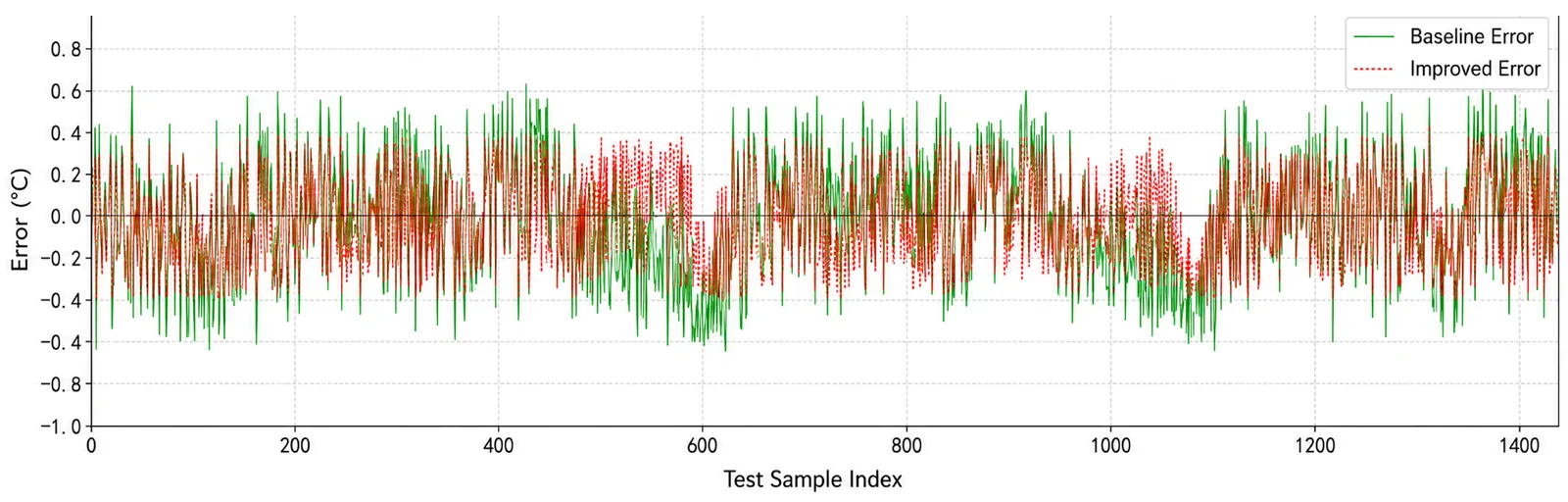
Comparison of Prediction Errors between The Basic BP-LSTM Model and The Attention-BP-LSTM Model.

Figure 9 further shows the prediction error distributions of the two models. From the error distribution, the error fluctuation range of Attention-BP-LSTM is further reduced, which shows that the optimized model outperforms the basic BP-LSTM model in both prediction accuracy and robustness. In particular, in intervals with rapid temperature changes or evident local fluctuations, the optimized model shows a more pronounced error suppression effect. This suggests that the attention mechanism helps the model focus on key temporal information, and Dropout regularization effectively alleviates overfitting during training, thereby enhancing the generalization ability of the model.

For a clearer quantitative comparison, RMSE, MAE, $R^{2}$ and Max Error were used to evaluate the prediction performance of different models. To reduce the influence of random initialization, each learning-based model was run ten times under the same data split and training settings, and the results are reported as the mean ± standard deviation. In addition to BP, LSTM, BP-LSTM, and the proposed Attention-BP-LSTM model, a simple persistence prediction method and a Transformer model were also included for comparison. The persistence method directly uses the current temperature as the predicted value for the next sampling point, while Transformer serves as a representative sequence prediction model based on the self-attention mechanism. Since the persistence prediction method does not involve model training or random initialization, its standard deviation is reported as 0.000 under the same test set. The comparison results are listed in Table 2.

To reduce the influence of random initialization, each neural network model was independently trained 10 times using different random seeds.

As shown in Table 2, the Attention-BP-LSTM achieved the best overall prediction performance, with an RMSE of 0.2227 ± 0.0102 °C, an MAE of 0.1769 ± 0.0065 °C, and an R^2^ of 0.9944 ± 0.0005. Compared with the Basic BP-LSTM, the RMSE and MAE were reduced by 80.83% and 79.87%, respectively, indicating that the optimized structure and attention mechanism substantially improved prediction accuracy. The Attention-BP-LSTM also outperformed the Transformer model, reducing RMSE and MAE by 28.75% and 24.64%, respectively. Although its maximum error was close to that of the Persistence baseline, its lower RMSE and MAE demonstrate better overall error control and more stable prediction performance.

In addition, Attention-BP-LSTM achieved the lowest average RMSE and MAE and the highest R^2^ among all trainable models. Compared with Basic BP-LSTM, RMSE and MAE were reduced by 80.83% and 79.87%, respectively. Compared with Transformer, RMSE and MAE were reduced by 28.75% and 24.64%, respectively. Although its maximum error was close to that of the Persistence baseline, Attention-BP-LSTM achieved lower overall RMSE and MAE, indicating better global prediction accuracy while maintaining acceptable worst-case error control. Overall, the performance of the models shows a gradual improvement from the single model to the hybrid model and then to the optimized model. The Attention-BP-LSTM model achieves higher accuracy and stability in the task of predicting axle-box bearing temperature in the next 3 min, and can provide more reliable temperature prediction results for bearing condition monitoring and fault warning.

## 5. Conclusions

This paper proposes a high-speed train axle-box bearing temperature prediction method based on time-delay low-rank sparse reconstruction and Attention-BP-LSTM. First, linear interpolation, time-delay low-rank sparse decomposition, and Z-score normalization are applied to preprocess the original axle temperature data, which improves data integrity and reliability. Then, an Attention-BP-LSTM model is constructed. The model uses LSTM to extract temporal dependence features from the temperature sequence, employs the attention mechanism to strengthen the representation of key historical information, and incorporates Dropout regularization to improve generalization. The experimental results show that the proposed model achieved the best overall performance, with an RMSE of 0.2227 ± 0.0102 °C, an MAE of 0.1769 ± 0.0065 °C, and an R^2^ of 0.9944 ± 0.0005. Specifically, compared with Basic BP-LSTM, the RMSE and MAE were reduced by 80.83% and 79.87%, respectively; compared with Transformer, they were reduced by 28.75% and 24.64%. These results verify that the combination of data reconstruction and model optimization effectively improves the accuracy and stability of short-term axle-box bearing temperature prediction.

## Figures and Tables

**Table 1:** Main Parameters of Attention-BP-LSTM Model.

Parameter	Value
Input window size	w
Prediction step	1
LSTM hidden units	64
BP hidden neurons	32
Dropout rate	0.3
Learning rate	0.001
Batch size	32
Epochs	100
Optimizer	Adam
Loss function	MSE

**Table 2:** Prediction Performance Metrics of Different Models.

Module	RMSE (°C)	MAE (°C)	$R^{2}$	Max Error (°C)
BP neural network	1.5489 ± 0.8802	1.2438 ± 0.6892	0.6499 ± 0.4976	4.1589 ± 1.8159
LSTM neural network	2.2763 ± 1.1181	1.6418 ± 0.9665	0.2870 ± 0.7295	6.5025 ± 1.8278
Basic BP-LSTM	1.1615 ± 0.2527	0.8784 ± 0.2229	0.8410 ± 0.0744	3.7212 ± 0.5382
Attention-BP-LSTM	0.2227 ± 0.0102	0.1769 ± 0.0065	0.9944 ± 0.0005	0.8060 ± 0.2030
Transformer-base	0.3125 ± 0.0264	0.2347 ± 0.0171	0.9889 ± 0.00019	1.3393 ± 0.1455
Persistence	0.2599 ± 0.000	0.2103 ± 0.0000	0.9924 ± 0.0000	0.8005 ± 0.0000

## Data Availability

The original contributions presented in this study are included in this article; further inquiries can be directed to the corresponding author.
